# A Micromachined Picocalorimeter Sensor for Liquid Samples with Application to Chemical Reactions and Biochemistry

**DOI:** 10.1002/advs.202003415

**Published:** 2021-01-12

**Authors:** Jinhye Bae, Juanjuan Zheng, Haitao Zhang, Peter J. Foster, Daniel J. Needleman, Joost J. Vlassak

**Affiliations:** ^1^ Department of NanoEngineering University of California San Diego La Jolla CA 92093 USA; ^2^ John A. Paulson School of Engineering and Applied Sciences Harvard University Cambridge MA 02138 USA; ^3^ Physics of Living Systems Department of Physics Massachusetts Institute of Technology Cambridge MA 02139 USA; ^4^ Department of Molecular and Cellular Biology Harvard University Cambridge MA 02138 USA; ^5^ Center for Computational Biology Flatiron Institute New York NY 10010 USA

**Keywords:** biosensors, calorimetry, Seebeck effect, thermoelectric effect, thermopiles

## Abstract

Calorimetry has long been used to probe the physical state of a system by measuring the heat exchanged with the environment as a result of chemical reactions or phase transitions. Application of calorimetry to microscale biological samples, however, is hampered by insufficient sensitivity and the difficulty of handling liquid samples at this scale. Here, a micromachined calorimeter sensor that is capable of resolving picowatt levels of power is described. The sensor consists of low‐noise thermopiles on a thin silicon nitride membrane that allow direct differential temperature measurements between a sample and four coplanar references, which significantly reduces thermal drift. The partial pressure of water in the ambient around the sample is maintained at saturation level using a small hydrogel‐lined enclosure. The materials used in the sensor and its geometry are optimized to minimize the noise equivalent power generated by the sensor in response to the temperature field that develops around a typical sample. The experimental response of the sensor is characterized as a function of thermopile dimensions and sample volume, and its capability is demonstrated by measuring the heat dissipated during an enzymatically catalyzed biochemical reaction in a microliter‐sized liquid droplet. The sensor offers particular promise for quantitative measurements on biological systems.

## Graphical abstract

A highly sensitive chip calorimetric sensor that is capable of resolving picowatt‐level heat dissipation using low‐noise thermopiles is developed. Four references are used to eliminate any effect of ambient temperature changes; a hydrogel‐lined enclosure minimizes sample evaporation without impact on the thermal mass of the sensor. The sensor is demonstrated for the oscillating Briggs–Rauscher reaction and the enzymatic hydrolysis of ATP.

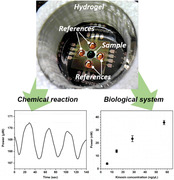

## 1 Introduction

Calorimetry is used to probe changes in the physical state of a system by measuring the heat exchanged between the system and its environment as a result of chemical reactions or phase transitions in the system. The technique is an essential tool in many fields, ranging from basic sciences such as biochemistry^[^ ^1^ ^]^ and high‐energy physics^[^ ^2^ ^]^ to the development of pharmaceuticals.^[^ ^3^, ^4^ ^]^ For example, isothermal titration calorimetry (ITC) and differential scanning calorimetry (DSC) are used extensively for the thermodynamic characterization of molecular binding and for the study of the internal structure of biomolecules in life science research. These techniques typically require relatively large sample volumes, as well as long stabilization and measurement times. Unfortunately, these techniques are not sensitive enough for measurements on single cells.

The development of microfabrication and microfluidics techniques has given rise to a class of chip calorimetry sensors that address many of these issues. These sensors rely on a variety of thermal sensing techniques, including thermocouple probes,^[^ ^5^, ^6^ ^]^ thin‐film thermocouples^[^ ^7^, ^8^ ^]^ and thermopiles,^[^ ^9^, ^10^, ^11^ ^]^ fluorescence,^[^ ^12^, ^13^ ^]^ thermistors,^[^ ^14^, ^15^, ^16^ ^]^ and infrared (IR) thermography.^[^ ^17^, ^18^ ^]^ Miniaturization of the sensors has reduced the required sample volume and sensor response time. In biology, these chip calorimeters provide the ability to characterize enthalpy changes associated with biological and chemical phenomena without the need for labeling or additional sample preparation, such as analyte immobilization.^[^ ^19^, ^20^ ^]^ They provide noninvasive and real‐time sensing, but the difficulty of handling microscale liquid samples and their relatively low thermal sensitivity remain critical challenges.

Measurements on microscale liquid samples are typically performed in one of two ways, using either an open or a closed‐chamber configuration on the calorimetry sensor. A sensor with an open‐chamber configuration requires placement of the liquid sample directly on the sensing area of the sensor, for instance, by pipetting^[^ ^21^, ^22^, ^23^, ^24^, ^25^ ^]^ or ink‐jet deposition.^[^ ^10^ ^]^ Calorimeter chips with an open‐chamber configuration typically have good thermal sensitivity, but the open‐chamber configuration allows evaporation of aqueous samples and makes it difficult to control the sample droplet. Torres et al. reported a chip calorimeter with an open‐chamber configuration that partially overcomes these drawbacks^[^ ^24^ ^]^ by integrating an electrowetting system to isothermally merge droplets on the sensor. This approach enables measurement of fast binding reactions, but the sensitivity of the sensor is only on the order of 100 nW because of noise in the semiconductor thermopile. The other method for handling microscale liquid samples relies on microfluidic channels to dispense a sample into a closed chamber on the calorimetry sensor.^[^ ^9^, ^26^, ^27^ ^]^ This approach eliminates sample evaporation and reduces destabilization of the sample during the measurement because of environmental factors, but the microfluidic channels increase the thermal mass of the sensor and may lead to enhanced heat loss to the ambient. As a result, these sensors tend to have lower sensitivity than sensors with an open‐chamber configuration. Furthermore, they may not be suitable for high‐viscosity biological samples that would be difficult to dispense using microfluidics. In an effort to reduce heat loss from the sample, Lee et al. developed a closed‐chamber calorimetry sensor designed to operate in vacuum.^[^ ^11^ ^]^ This sensor uses a metal thermopile to measure temperature differences as small as 500 µK, which corresponds to a sensitivity of 4.2 nW. Another, more recent, device also relies on vacuum to reduce heat loss to the environment, but uses the resonance frequency of a silicon beam instead of a thermopile to detect temperature changes.^[^ ^28^ ^]^ This sensor can detect temperature variations on the order of 1.6 mK. The authors demonstrated that they could detect brown fat cells, an especially thermogenic type of cell, in a microfluidic channel, but did not report the power sensitivity of the device. There is growing interest in measuring the metabolic rate of single cells as a function of environmental factors or at different stages of development. Microfluidic calorimeters with 0.2 nW sensitivity, which represents a more than tenfold enhancement over the previous record, were reported just recently.^[^ ^16^, ^29^ ^]^ Considering that the metabolic power of a single mammalian cell is typically on the order of 10–100 pW,^[^ ^30^ ^]^ it is evident that this requires calorimeter sensors with a resolution of a few picowatts or better.

Here, we describe a micromachined calorimeter sensor that uses a series of low‐noise metal thermopiles to measure minute temperature differences between a liquid sample and several coplanar references. The sensor has a resolution on the order of tens of picowatt and relies on a hybrid approach for the handling of liquid samples that combines the advantages of open and closed chambers through use of a noncontact hydrogel enclosure. The sample of interest is loaded onto the sensor by direct pipetting resulting in a discrete sample droplet. This droplet is covered by a thin oil layer and enclosed inside a small hydrogel‐lined chamber to ensure long‐term stability. Prior to the measurement, the hydrogel is equilibrated with water at the same temperature as the sample so that it maintains a water partial pressure inside the chamber that is close to the vapor pressure of water at that temperature, thus reducing the driving force for evaporation. It does so without direct contact with the sample, and therefore, without significantly increasing heat loss from the sample, an essential feature to maximize sensor sensitivity. In the absence of physical confinement, the surface wetting properties of the sensor are tailored to ensure that the sample remains in the sensing area of the sensor. To achieve picowatt resolution, the sensor relies on four metal thermopiles that are designed to minimize the noise equivalent power using an analytical model of the temperature distribution surrounding the sample. The combination of low‐noise thermopiles with the hybrid sample handling technique results in an unparalleled sensor sensitivity. The sensor uses four references in a coplanar arrangement to eliminate the effects of external temperature gradients, allowing measurements over the span of hours. The sensor has an integrated heating element that allows direct calibration of the thermopiles in terms of dissipated power, thus obviating the need for a separate calibration of the temperature response of the thermistors.

In the following, we describe the sensor in detail and evaluate its performance for several different layouts. We measure the thermal response of the sensor using an ionic liquid as a sample and evaluate the evaporation rate of aqueous samples. We then demonstrate the sensor by measuring the heat output of the well‐known Briggs–Rauscher oscillating chemical reaction and of an enzymatically catalyzed biochemical reaction.

### 1.1 Design of Sensor

**Figure 1:**
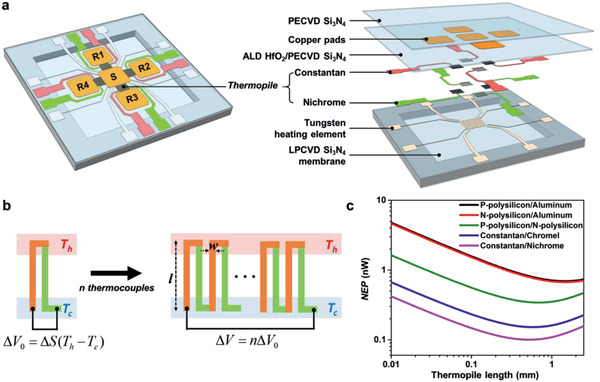
Pico‐calorimeter design. a) Overall sensor layout including a sample (S) and four reference (R1 to R4) pads; sequence of layers within sensor. b) Thermoelectric effect for a thermocouple and thermopile. c) The noise equivalent power as a function of thermopile length for different material pairings (*n* = 51, *h*_TP_ = 500 nm, *w* = 5 µm, *L*_o_ = 0.525 mm, *B* = 1 Hz, *T* = 300 K, material properties are listed in the Supporting Information).

**Figure** **1**a shows the overall layout of the calorimetric sensor. It consists of a thin silicon nitride membrane with a central pad for the sample (S in Figure 1a), surrounded by four reference pads (R1 to R4 in Figure 1a). The sample pad and the reference pads consist of a thin layer of copper embedded within the silicon nitride membrane to ensure temperature uniformity across each pad. By placing the reference pads on the membrane as opposed to the substrate, the temperature differences between the sample and the references arise mainly because of different physical or chemical processes taking place in either. Four coplanar references are used instead of just one to quantify and account for any in‐plane temperature gradients that may arise because of ambient conditions. Conversely, if the roles of sample and references are reversed, it is possible to perform measurements on four samples simultaneously, but at the expense of reduced measurement stability. The temperature differences between the sample and the references are measured directly using thermopiles. The sample area has a built‐in tungsten heating element in a four‐point measurement configuration to calibrate the sensor. Using this heating element, the voltage outputs of the thermopiles can be calibrated directly to the power dissipated in the sample area.

Thermopiles consist of a number of thermocouples connected in series (Figure 1b). The resolution of a thermopile‐based calorimeter sensor is determined by the signal‐to‐noise ratio of the thermopile, which depends mainly on the materials used in the thermopile and its geometry . To maximize the resolution of the calorimeter sensor, we minimize the noise equivalent power (NEP) of the thermopile, which can be written as the ratio of the Johnson noise (*V*_JN_) to the thermopile responsivity (Σ) (see the Supporting Information)

(1) $$\begin{array}{c} \mathrm{NEP}\;=\frac{V_{\mathrm{JN}}}{\Sigma }\;=\;16\pi \lambda_{g}\sqrt{k_{B}TB}\frac{\sqrt{\rho_{A}+\rho_{B}}}{S_{B}-S_{A}}\frac{\sqrt{lw/h_{\mathrm{TP}}}}{1-\exp\left(-\sqrt{\frac{\lambda_{g}}{kh_{\mathrm{TP}}L_{0}}}l\right)} \end{array}$$

based on a simple analytical thermal model of the sensor. Figure 1c shows the NEP of the thermopile as a function of thermopile length. It is evident that a thermopile with a length of approximately 0.5 mm and that consists of a combination of constantan and nichrome provides the best possible NEP.

### 1.2 Computational Thermal Model

**Figure 2:**
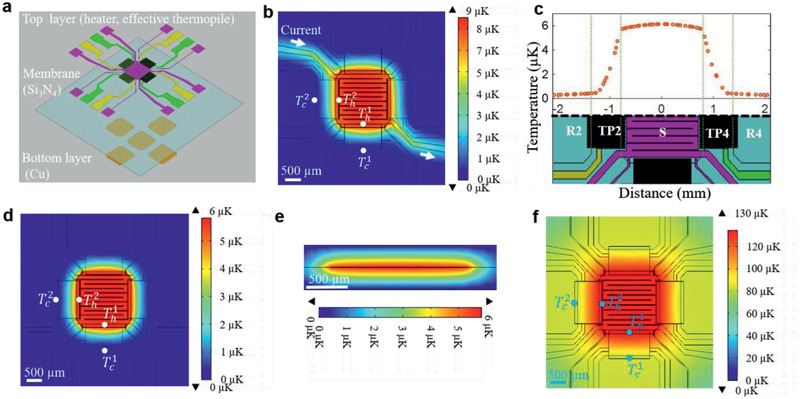
3D thermal model and calibration. a) Schematic of the finite element model of the sensor; the dimensions are those of sensor 22. All temperatures shown in the figures represent temperature differences from the ambient temperature. b) Temperature distribution within the sensor when a current of 10 µA is applied to the built‐in heating element, corresponding to a nominal power of 5.166 nW dissipated in the sample area. $(T_{h}^{1}\;-T_{c}^{1}=6.76\;\mu K,\;\mathrm{and}\;T_{h}^{2}\;-T_{c}^{2}=6.57\;\mu K$). c) Temperature distribution along a horizontal line through the center of the sample area when a power of 5.166 nW is dissipated in the sample area. The temperature is the temperature difference between the local temperature and the ambient temperature. d) Temperature distribution within the sensor when a power of 5.166 nW is applied directly to the sample area as would be the case in an actual measurement on an exothermic system $\left(T_{h}^{1}\;-T_{c}^{1}=\;5.40\;\mu K,\;\mathrm{and}\;T_{h}^{2}\;-T_{c}^{2}=5.42\;\mu K\right)$. e) Temperature distribution within a perpendicular plane through the center of the sensor when a power of 5.166 nW is applied to the sample area. f) Temperature distribution within the sensor when a power of 5.166 nW is applied directly to the sample in vacuum $\left(T_{h}^{1}\;-T_{c}^{1}=49.5\;\mu K,\;\mathrm{and}\;T_{h}^{2}\;-T_{c}^{2}=50.01\;\mu K\right)$.

A more detailed 3D thermal model of the sensor (**Figure** **2**a) was constructed to quantitatively analyze the temperature distribution within the sensor and surrounding area using a commercial finite element package, COMSOL Multiphysics. The in‐plane dimensions and the film thicknesses used in the model correspond to those of sensor 22 (**Table** **1**), which was also used in the majority of measurements in this study. Only heat loss by conduction through air and through the sensor membrane is considered. Under typical operating conditions, heat loss by natural convection is completely negligible given the very small value of the Grashof number (Gr ≈ 10^−6^ for Δ*T* ≈ 10^−6^ K), as is any heat loss by radiation. The temperature of the edge of the sensor was kept at an ambient temperature of 293.15 K, and the sensor was located inside a 0.51 mm tall enclosure, which was also kept at 293.15 K. The height of the enclosure was selected to bring the temperatures as determined from the simulations in agreement with experimental results, assuming ideal Seebeck behavior for the thermopiles. Instead of resolving the individual legs of the thermopiles, the thermopiles were homogenized to reduce the complexity of the model and computation time. All material parameters used in the model are listed in Table S1 (Supporting Information). The results of the simulations are summarized in Figure 2b–f. According to the finite element model, the thermal conductance of sensor 22 is 9.5 × 10^−4^ W K^−1^ under ambient conditions, and 1.0 × 10^−4^ W K^−1^ in vacuum. The experimental value of the thermal conductance of this sensor under ambient conditions is 9.67 × 10^−4^ W K^−1^.

**Table 1:** Design parameters of calorimetry sensors

Sensor	Width of sample pad [µm]	Thermopile length *L* [µm]	Thermopile line width *w* [µm]	Number of thermocouples *n*
22, 21	1500	525	5	51
32	1500	525	20	13
42	1500	525	20	9
52	1500	525	20	5
63	700	525	5	20
72	700	525	20	5
11	700	1500	5	20
36	700	1500	20	5
56	700	2000	5	20
66	700	2000	20	5

To simulate the sensor calibration procedure, power was generated in the sample area by passing a current through the tungsten heating element in the sample area. The steady‐state results obtained from the finite element model are shown in Figure 2b. It is evident from the figure that a uniform temperature distribution develops within both the sample and the reference areas thanks to the presence of the Cu layers in these areas. As desired, most of the temperature drop occurs across the thermopiles (Figure 2c). A slight temperature nonuniformity develops in the area surrounding the lines that supply the current to the heating element. This nonuniformity is the result of the heat dissipated in the current lines and leads to a slightly larger temperature difference across the thermopiles than arises during an actual measurement when only the sample dissipates heat. This effect is illustrated in Figure 2d, which show the temperature distribution in the sensor as a result of a sample dissipating heat at the same nominal power as the heating element during the calibration. Because there is no current flowing through the heating element in Figure 2d, the temperature nonuniformity associated with the heating lines is absent in this figure. The actual temperature difference during such a measurement (Figure 2d) is 79.9% of the temperature difference during the calibration (Figure 2b), and the responsivity of the sensor obtained from the calibration process using the in situ heating element needs to be corrected accordingly. The corresponding temperature difference for the sensors with the smaller sample area is 80.2%. This correction factor is a function of the geometry of the sensor and the thermal properties of the various components of the sensor only and should therefore be predicted quite accurately by the finite element model, provided the materials properties are well known. Both for the calibration and for actual measurements, there is a difference in the temperature drop across the thermopiles as a result of the nonsymmetric layout of the tungsten heating element. This difference is very small and typically less than the experimental error in the measurements. Figure 2e shows the temperature distribution within a vertical plane through the center of the sensor. It is evident that the temperature distribution is not quite spherically symmetric as assumed in the analytical thermal model because of the proximity of the enclosure. As a result, the analytical model overestimates the temperature differences that develop in the sensor. Because most of the heat loss during a measurement occurs by conduction through air, the responsivity of the sensor can be increased significantly by making measurements in vacuum.^[^ ^11^, ^31^ ^]^ While measurements in vacuum may not be suitable for biological samples, they may be perfectly acceptable for samples with low vapor pressure. Figure 2f shows the temperature distribution for a typical measurement performed in vacuum. It is clear from the figure that the temperature difference induced by the sample is significantly larger than in for a measurement performed in air, albeit at the expense of a slightly reduced temperature uniformity. The simulation results indicate that the responsivity in vacuum is improved by a factor of 9.15 for the sensors with the larger sample area and 7.29 for those with the smaller area (Table 1).

### 1.3 Fabrication and Measurement Setup

Arrays of sensors were fabricated using various microfabrication techniques (Figure S1, Supporting Information). First, a 600 nm silicon nitride (Si_3_N_4_) coating was grown on a Si substrate using low‐pressure chemical vapor deposition (LPCVD). Then, a tungsten heating element with a thickness of 120 nm and four constantan/nichrome thermopiles with a thickness of 500 nm were fabricated on top of the LPCVD Si_3_N_4_ coating. Tungsten, nichrome, and constantan coatings were deposited by magnetron sputtering and patterned using standard lithography and lift‐off techniques. The heating element and the thermopiles were subsequently coated with 30 nm of HfO_2_ and 300 nm of Si_3_N_4_ using atomic layer deposition (ALD) and plasma‐enhanced chemical vapor deposition (PECVD), respectively. These dielectric layers served to electrically isolate the heating elements and thermopiles and allowed the deposition of a 1 µm layer of Cu within the sample and reference areas to enhance the temperature uniformity within these areas (Figure 2b–d). After depositing 100 nm of PECVD Si_3_N_4_, the sensors were made freestanding by deep reactive ion etching. Finally, the entire surface of the sensor array was treated with perfluorooctyltrichlorosilane (PFOTS) to create a low‐energy surface. This treatment resulted in a hydrophobic and oleophobic surface that prevented the merging of the oil‐coated sample and reference droplets after loading.

**Figure 3:**
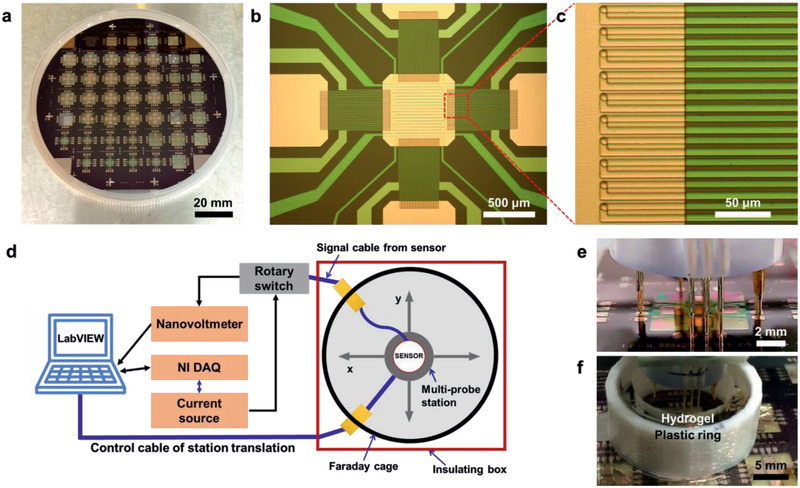
Sensor array and measurement setup. a) Sensor array. b,c) Optical microscopy images of the fabricated sensor and thermopile. d) Schematic illustration of the entire data acquisition system and measurement setup. e) Probes contacting sensor without hydrogel‐lined enclosure in place. f) Probes contacting sensor with enclosure in place.

Sensors with different thermopile length, line width, and number of thermocouples were fabricated, as well as sensors with different sizes of the sample areas, as enumerated in Table 1. **Figure** **3**a shows an optical image of a finished array. The transparent areas correspond to the freestanding Si_3_N_4_ membranes that support the sensors. The sensing area in the center of a sensor is shown in Figure 3b. The copper pads, the serpentine heating element, and the thermopiles are readily discerned. Figure 3c shows an area where a thermopile overlaps with the Cu pad in the sample area, with the thermopile junctions clearly visible. Parallel with the sensor fabrication process, a data acquisition (DAQ) system and measurement setup were developed. The DAQ system was designed to maximize signal‐to‐noise ratio and to operate at a noise level lower than the Johnson noise of the sensors.^[^ ^32^ ^]^ The measurement setup consists of an automated probe station with a camera to facilitate probe alignment and a small hydrogel‐lined enclosure (Figure 3d–f). In a typical measurement, liquid samples and references are loaded by direct pipetting. To prevent evaporation, sample and reference droplets are coated with a thin layer of oil and sealed inside the hydrogel‐lined enclosure. It takes 15–20 min for the water partial pressure in the enclosure to equilibrate.

### 1.4 Sensor Calibration

**Figure 4:**
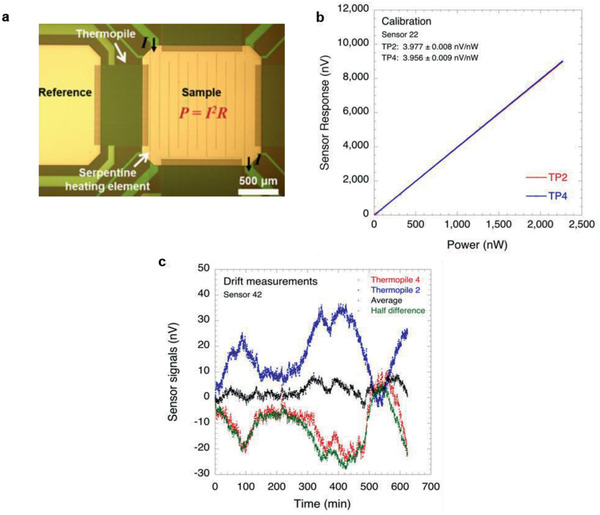
Sensor calibration. a) Optical microscopy image of the sample area illustrating the current flow through the heating element during the calibration. b) Response of sensor 22 as a function of power applied to the heating element. c) Response of sensor 42 over a period of ≈11 h without power applied to the sensor heating element. The average of the output of the two colinear thermopiles represents the sensor signal and is evidently much less sensitive to thermal drift than the signal from individual thermopiles. A 100‐reading moving average filter was used for the measurements.

The calorimetric sensors were calibrated using the built‐in heating capability. This approach has the advantage that the input power can be precisely controlled by varying the current through the heating element (**Figure** **4**a). The sensor responsivity is then determined directly from the response of the thermopile as a function of input power. All measurements were performed in air on sensors without samples. Values for vacuum conditions were obtained by dividing the ambient calibration values by a factor of 9.15 or 7.29, obtained from the finite element models, depending on the size of the sample sensor area. The results are summarized in **Table** **2**. Figure 4b shows typical results for two individual thermopiles on sensor 22 (see Table 1). It is evident from the figure that the response of the sensors is linear over the entire range of input power. The resolution of the sensors depends on the responsivity of the sensor and the noise (see the Supporting Information) in the measurements and may be determined from the NEP listed in Table 2. When the four thermopiles are connected in series, the responsivity increases by a factor of 4, while the noise increases only by a factor of 2. For instance, if the four thermopiles in sensor 63 are connected in series, the responsivity in vacuum of the sensor is 133.4 V W^−1^ and the total noise is 3.74 nV (see Table 2). Combining both results leads to a resolution in vacuum of ≈28 pW. The corresponding resolution under ambient conditions is 200 pW. These values of the resolution can be further improved for slowly varying signals by accepting a smaller bandwidth. A more detailed discussion can be found in Section S5 (Supporting Information).

**Table 2:** Characteristics of the various sensor designs. Single‐thermopile responsivities were measured using the internal heating capability. The total noise includes both Johnson noise and instrument noise and was calculated using Equation (S9) (Supporting Information) for a single thermopile. The NEP in air was calculated using the total noise at a bandwidth of 0.02 Hz, the NEP in vacuum was obtained by dividing the NEP in air by a correction factor of 9.15 and 7.29 depending on the size of the sensor sample area

	Single thermopile						Four thermopiles in series			
Sensor no.	TP resistance [kΩ]	Responsivity [V W^−1^]	Johnson noise [nV]	Total noise [nV]	NEP in air [nW]	NEP in vacuum [pW]	Johnson noise [nV]	Total noise [nV]	NEP in air [nW]	NEP in vacuum [pW]
22	20.6	3.967 ± 0.008	2.64	2.78	0.70	76	5.28	5.35	0.34	37
32	1.42	1.110 ± 0.003	0.69	1.11	1.00	109	1.38	1.64	0.37	40
42	0.98	0.709 ± 0.001	0.58	1.04	1.47	160	1.15	1.44	0.51	55
52	0.58	0.434 ± 0.001	0.44	0.98	2.26	246	0.89	1.24	0.71	78
63	9.80	4.573 ± 0.002	1.82	2.02	0.44	61	3.64	3.74	0.20	28
72	0.58	1.100 ± 0.002	0.44	0.98	0.89	122	0.89	1.24	0.28	39
11	19.56	4.642 ± 0.004	2.57	2.71	0.58	80	5.14	5.22	0.28	39
36	1.45	1.158 ± 0.003	0.70	1.12	0.97	133	1.40	1.65	0.36	49
56	30.72	4.643 ± 0.002	3.22	3.34	0.72	99	6.44	6.50	0.35	48
66	1.95	1.156 ± 0.001	0.81	1.19	1.03	141	1.62	1.84	0.40	55

Figure 4c shows the signals obtained from two individual thermopiles that are colinear with the sample area over a period of ≈11 h without any power applied to the heating element. Along with these signals, the average and half difference of the thermopile signals are also shown. The difference signal is proportional to the in‐plane temperature gradient in the direction of the thermopiles, while the average of the two thermopiles yields the sensor signal corrected for the in‐plane temperature gradient. It is evident from the figure that the stability of the average signal is significantly improved compared to the signals from the individual thermopiles or the difference signal. In Figure 4c, the maximum temperature difference between sample and references over a period of 11 h is ≈55 µK. The effect of this temperature difference on the measurements is largely eliminated through the use of two colinear references. The small amount of drift that remains in the sensor signal may be caused by drift in the data acquisition equipment as a result of changes in the ambient temperature and possibly by a small difference in the responsivity of the individual thermopiles (typically less than 0.5%). Only two thermopiles were used in these measurements because the current configuration of the data acquisition equipment only allows simultaneous acquisition of two signals.

A detailed analysis of the results in Table 2 demonstrates that, for a given thermopile length, the responsivity of the sensors increases linearly with the number *n* of thermocouples in the thermopile divided by sample size *a*, as expected based on the simple thermal model. For a fixed value of *n*/*a*, the responsivity increases and eventually saturates with thermopile length, again in accordance with the thermal model. When considering both the measurement noise and sensor responsivity, however, the conclusions are different. For a given thermopile width and number of thermocouples, the value of the NEP increases with thermopile length. While there are no data for sensors with thermopile lengths shorter than 525 µm, it is evident that the NEP must diverge as the thermopile length approaches zero, i.e., there exists a thermopile length that minimizes the NEP and hence optimizes the resolution of the sensor. For a given thermopile length, the NEP decreases with increasing number of thermocouples in the thermopile provided the line width is held constant.

### 1.5 Measurements on Chemical and Biological Systems

Since the calorimetry sensor was developed mainly for the study of chemical and biological systems, it is important to understand the behavior of the sensor in conjunction with liquid samples. Experiments involving four different liquid systems were performed: heating of an ionic liquid, spontaneous evaporation of water droplets, enthalpy production during an oscillating aqueous chemical reaction, and enthalpy production during ATP hydrolysis by a biological enzyme.

**Figure 5:**
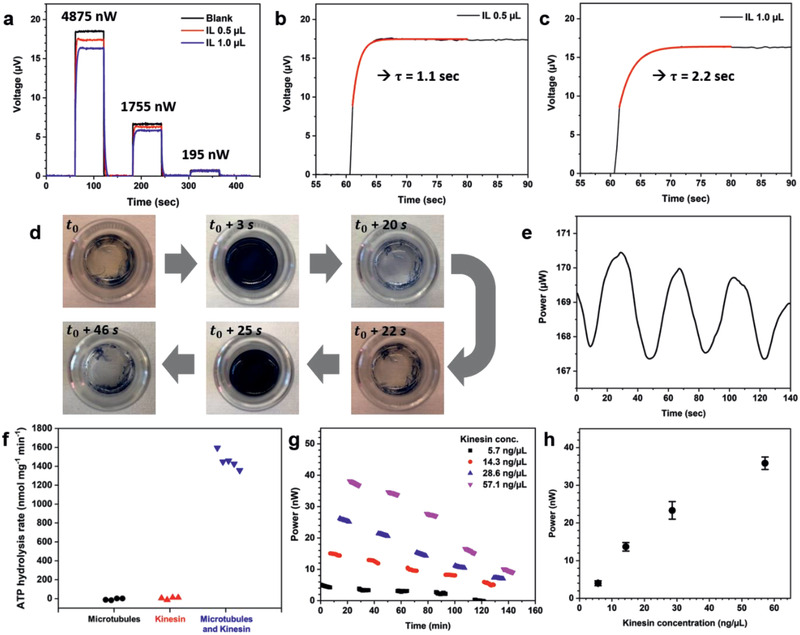
Calorimetry measurements on liquid samples. a) Thermoelectric response of the sensor with different volumes of ionic liquid; b,c) response time constant with different liquid volumes: b) *τ* = 1.1 s when the volume is 0.5 µL and c) *τ* = 2.2 s when the volume is 1.0 µL. Briggs–Rauscher reaction: d) Photographs of color oscillations and e) measurement of enthalpy production with the calorimetric sensor. ATP hydrolysis: f) Measured ATP hydrolysis rate for samples containing microtubules, kinesin, or both microtubules and kinesin. g) Power output (nW) as a function of time with different kinesin concentration. h) Average power outputs for the first hour with increasing kinesin concentration in the range of 5.7–57 ng µL^−1^. The error bars in (h) represent the standard deviation of the measurements over a period of 1 h. Sensor 22 was used for all measurements in (a)–(c) and (f)–(h); sensor 32 was used for the measurements in (e). A 10‐reading moving average filter was applied to the measurements in (e)–(h), no filter was applied used for the data in (a)–(c).

In a first experiment, droplets of an ionic liquid (IL, 1‐butyl‐3‐methylimidazolium hexafluorophosphate) were used to analyze the response of the sensor as a function of the liquid sample volume and applied power. Since the evaporation of ionic liquids is negligible at room temperature, the response time can be obtained directly from the sensor response. As illustrated in **Figure** **5**a, three different currents, corresponding to power levels of 4875, 1755, and 195 nW, were applied to a blank sensor, as well as to a sensor loaded with two different volumes (i.e., 0.5 and 1.0 µL) of ionic liquid.

According to Equation (S2) (Supporting Information), the temperature difference between the sample and the ambient increases linearly with power and decreases inversely with sample size. This trend is indeed observed in Figure 5a, although the precise definition of sample radius *a* is not evident given that heat loss occurs from both the sample pad area and the surface of the sample. Thus, the sample radius used in Equation (S2) (Supporting Information) may be regarded as an effective sample radius that depends on the size of both the sample droplet and the heated sample area, and that accounts for the approximate nature of Equation (S2) (Supporting Information). While the effect of sample size on the response of the sensor is quite small, it should be taken into account for precise measurements. This can be done by performing the sensor calibration in the presence of a sample of appropriate size or through use of the effective sample radius.

Next, we examine the time constant (*τ*) of the response for different volumes of ionic liquid. A simple exponential fit of the output voltage of the sensor as a function of time shows that the response time is independent of applied power but increases linearly with sample volume over effective sample radius, as one would expect based on a simple lumped thermal model (see Figure S4, Supporting Information). The response time of a sensor with a typical sample is on the order of a few seconds, whereas the response time of a blank sensor is ≈10 ms as a result of its much smaller thermal mass. In either case, the sensor reaches the steady state in a very short period of time making it suitable for measurements of time‐varying events with time constants greater than a few seconds.

To evaluate the effect of evaporation of an aqueous sample or reference, calorimetric measurements were performed using two 0.5 µL droplets of water. One of the droplets was placed in the sample area and the other droplet was placed in one of the reference areas. The droplet in the sample area was coated with 0.5 µL of mineral oil to slow down evaporation. The results are shown in Figure S5 (Supporting Information). The graph shows two distinct phases in the measurement: Initially, the signal is positive and decreases slowly with time. Approximately 180 min into the measurement, the signal decreases rapidly, becomes negative, and then again changes slowly in time. This behavior can be understood as follows: The bare reference droplet evaporates faster than the oil‐coated sample droplet resulting in a cooling of the reference area compared to the sample area. Once the reference droplet has completely evaporated, the cooling associated with evaporation of the sample droplet reverses the signal. The nearly constant signal during the two distinct phases of the measurement arises because the partial pressure inside the hydrogel cylinder that surrounds the sensor is close to the vapor pressure of water, and thus the overall evaporation rate is limited by the loss of water from the enclosure surrounding the sensor. If water loss from the enclosure were not limiting, by contrast, the calorimetry signal would decay quadratically instead. The evaporation rates of the droplets can be estimated by integrating the calorimetric signal to determine the enthalpies associated with the evaporation of the respective droplets. Extrapolating the calorimetric signal during the second phase and using it as a baseline for the signal during the first phase shows that full evaporation of the uncoated reference droplet required 1.04 J after the start of the measurement. Partial evaporation of the oil‐coated sample droplet required 0.57 J over the same time period. This result suggests that the oil‐coated droplet evaporates at approximately half the rate as the uncoated droplet. The effect of the oil coating on the evaporation rate is not as large as one might at first expect because evaporation of the uncoated droplet slows down significantly once the sensor is enclosed in the hydrogel enclosure, and the evaporation rate is controlled by transport of water from the enclosure to the ambient. In fact, the small difference in evaporation rate between the two droplets is a good demonstration of the effectiveness of the hydrogel enclosure in reducing evaporation, allowing measurements over long periods of time.

To illustrate use of the sensor in chemical reactions, the sensor signal was measured for the Briggs–Rauscher reaction, a well‐known oscillating chemical reaction.^[^ ^33^, ^34^, ^35^, ^36^ ^]^ Three solutions were prepared: Solution A (4 m hydrogen peroxide (J. T. Baker)); Solution B (0.2 m potassium iodate (Alfa Aesar) and 0.077 m sulfuric acid (VWR)); and Solution C (0.15 m malonic acid (Alfa Aesar), 0.02 m manganese sulfate monohydrate (VWR), and 1.1 mg mL^−1^ of starch (MP Biomedicals)). The three colorless solutions were mixed (1:1:1 by volume) to produce an oscillating reaction that showed a color cycle from amber to dark blue and then to colorless. (Figure 5d). The real‐time calorimetric signal was measured for a 1.5 µL droplet of the ABC mixture deposited on the sample pad with a 1.5 µL droplet of solution B used as a reference. As shown in Figure 5e, the measured voltage signal oscillated as a function of time. The peak‐to‐peak amplitude in Figure 5e was 2.5 µW corresponding to a temperature difference of 10.2 mK between the oscillating sample and the reference solution. A lumped thermal model shows that the measured power amplitude is reduced by a factor of 1.6 because of the oscillating nature of the signal. Consequently, the observed power amplitude is 2.6 W L^−1^, significantly lower than measured in bulk experiments, presumably because of the lack of stirring.^[^ ^37^ ^]^ Note that the time interval between peaks (i.e., crest‐to‐crest or trough‐to‐trough) in the calorimetric signal is 36 s, whereas the dark blue to dark blue cycle in Figure 5d was 22 s and the colorless to colorless cycle took 26 s. The difference in cycle time is not surprising given that the oscillation period of the reaction increases as the reaction proceeds,^[^ ^33^ ^]^ and the two measurements were not obtained simultaneously.

We next measured the heat dissipation during enzymatically catalyzed biochemical reactions in microliter‐sized liquid droplets. As a model enzyme, we used a kinesin motor protein because of its biological relevance and its use as a standard for measuring the ATP hydrolysis rates of motor proteins. Kinesins are ATPases, which in living cells use the energy derived from ATP hydrolysis to walk on microtubules and transport subcellular cargos throughout the cell. Kinesin heavy chain, the kinesin subunit containing the motor and microtubule‐binding domains, and reagents were purchased as part of a Kinesin ATPase Endpoint Assay kit (Cytoskeleton, Inc., Cat. No. BK053). Unless otherwise noted, samples were prepared as described in the kit instructions. Reactions were initiated by the addition of ATP (Sigma–Aldrich).

We first performed measurements confirming the ATP hydrolysis rate of kinesin heavy chain following the Kinesin ATPase Endpoint Assay protocol, which is adapted from the method of Kodama et al.^[^ ^38^ ^]^ Briefly, samples were allowed to hydrolyze ATP for 5 min, after which the reaction was terminated by the addition of the included CytoPhos reagent, which causes a change in absorbance at 650 nm that increases with free phosphate concentration. Each ATP hydrolysis reaction leads to a free phosphate, and the total amount of free phosphate is measured after the reaction is terminated by comparing the sample absorbance with a measured calibration curve (Figure S6, Supporting Information). The ratio between the amount of free phosphate and the time interval provides a measure of the underlying ATP hydrolysis rate. Consistent with previous reports,^[^ ^39^ ^]^ we confirmed that the measured ATP hydrolysis rate is strongly stimulated by the presence of microtubules and that kinesin heavy chain has an ATP hydrolysis rate per mg of kinesin of 1460  ± 90 nmol mg^−1^ min^−1^ in these conditions (Figure 5f).

Subsequently, we measured the heat dissipation as a function of kinesin concentration and time. Samples with a volume of 0.5 µL, each corresponding to a different kinesin concentration, were placed on the sample pad of the sensor, and a sample lacking kinesin was used as a reference. Samples were prepared as above, but with ATP added to a final concentration of 14.3 × 10^−3^ m and with the reaction termination step omitted. To diminish the effects of evaporation, both the reference and samples were covered with 0.5 µL of mineral oil before measurements were taken. The measured dissipation was found to increase monotonically with the amount of kinesin in the sample, and to gradually decrease over time for all samples (Figure 5g). For each sample, we next averaged the measured dissipation over the first hour of measurements. As expected, the dissipated power was found to increase approximately linearly with the amount of kinesin (Figure 5h). This is consistent with the ATP hydrolysis rate for kinesin being governed by Michaelis–Menten kinetics, where the total rate of ATP hydrolysis is limited by the amount of kinesin in the sample, i.e., a constant ATP hydrolysis rate per mg of kinesin.

## 2 Conclusions

We have developed an ultrasensitive micromachined calorimetric sensor, designed specifically for analyzing liquid samples with a volume on the order of 1 µL. The sample of interest and up to four references are supported by a Si_3_N_4_ membrane that isolates them thermally from the ambient. Evaporation of aqueous samples is minimized through use of a hydrogel‐lined enclosure that maintains the partial pressure of water inside the enclosure at the saturation level with minimal impact on the thermal mass of the sensor. Calorimetric measurements are then performed by measuring the temperature difference between the sample and up to four references using low‐noise thermopiles. The use of several references makes it possible to eliminate the effect of in‐plane temperature gradients that may arise during the measurement as a result of small changes in the ambient temperature, and the built‐in heating element allows for a direct calibration of the thermopile response to the power dissipated in the sample area. The materials used to fabricate the sensors and its geometry have been selected to minimize the noise equivalent power of the sensor, resulting in a sensor with a resolution on the order of 30–200 pW depending on the desired bandwidth and environmental conditions. This extraordinary sensitivity makes the sensor ideally suited for the study of nonequilibrium processes in biological systems, as illustrated by measurements of the heat produced by ATP hydrolysis in the kinesin/microtubule system. The ease of gas exchange facilitated by the small sample volume, fast temporal response, and the special handling of aqueous samples make this sensor particularly promising for direct measurements of metabolic rate at the single‐cell level. Single‐cell measurements would provide insight into the cell‐to‐cell heterogeneity of energy use, which is inaccessible from measurements at the population level. Recent papers measuring the heat dissipated from individual^[^ ^40^ ^]^ or small numbers^[^ ^41^ ^]^ of embryos have uncovered temporal oscillations in the thermal dissipation which correlate with the underlying cell cycle, highlighting the dynamic nature of cellular energy use. Additionally, the thermal dissipation from embryos early in development has been measured to range from 60 nW in zebrafish^[^ ^41^ ^]^ to 100s of nW in *Xenopus laevis* ^[^ ^40^ ^]^ and *Drosophila melanogaster*,^[^ ^42^ ^]^ well above the NEP of the sensors described here.

## 3 Experimental Section

##### Sensor Fabrication

The fabrication process started with a (100) Si wafer passivated with a 300 nm super low‐stress LPCVD silicon nitride coating (WRS Materials). To reduce buckling of the Si_3_N_4_ membranes, an additional 300 nm low‐stress silicon nitride film was grown using LPCVD (Tystar Furnace) (Figure S1a, Supporting Information). The heating element and the thermopiles were fabricated using standard lithography and lift‐off processes. First, a 1.5 µm thick photoresist (Shipley1813, Dows) was spin‐coated onto the substrate, exposed through a photomask to UV light with a wavelength of 405 nm, and developed in CD30 developer for 1 min. Next, 5 nm titanium and 120 nm tungsten were deposited using magnetron sputtering in a high‐vacuum chamber (ATC 1800; AJA International) after Ar sputtering for 3 min. Finally, the metal stack was patterned by lifting off the photoresist in acetone and isopropanol baths with sonication (Figure S1b, Supporting Information). This process was repeated two more times to fabricate the thermopiles, first to pattern a stack of 5 nm of titanium and 500 nm of nichrome, and then 5 nm of titanium and 500 nm of constantan (Figure S1c, Supporting Information). In a subsequent step, the heating element and the thermopiles were coated with 30 nm of hafnium oxide (HfO_2_) using ALD (Cambridge Nanotech Savannah 200 ALD system) and 300 nm of Si_3_N_4_ using PECVD (Nexx Cirrus 150) (Figure S1d, Supporting Information). Next, the sample and reference areas were delineated by sputter‐depositing and then patterning 5 nm of titanium and 1 µm of copper using the standard lift‐off process (Figure S1e, Supporting Information). In all metal depositions, the titanium film served as adhesion layer to prevent delamination of the metal coatings. To protect the sensor from oxidation and direct contact with liquids, a 100 nm Si_3_N_4_ capping layer was deposited using PECVD (Figure S1f, Supporting Information). After this step, the sensor was annealed at 450 °C for a period of 8 h in a vacuum furnace with a base pressure of 10^−7^ Torr to release stresses and enhance adhesion between different layers. To make the sensor freestanding, the backside of Si was selectively etched away by deep reactive ion etching (DRIE; SPTS Rapier DRIE) (Figure S1g, Supporting Information). In a final step, the surface of the sensor was treated with PFOTS (Oakwood Chemical) to make the surface both hydrophobic and oleophobic. This step ensured that both aqueous and oil droplets beaded up with large contact angle to prevent sample and reference droplets from spreading out over the thermopiles.

##### Data Acquisition System

All measurements were conducted inside a Faraday cage containing a custom‐built automated multiprobe *X*–*Y* translation stage (T‐LSM100A, Zaber Technologies Inc.) and a vertical piezo‐stage (AG‐LS25V6; Newport Corp.). The Faraday cage was surrounded by an insulating box to improve temperature stability during the measurement. The entire setup was housed in a room where the temperature was controlled to within 0.5 °C. A 12‐pin probe head was mounted on the vertical piezo‐stage, and two digital microscopes were used to assist with the alignment between the sensor contacts and the probe and with the loading of samples and references. For the calibration of the sensors, a current was applied to the built‐in heating element in the sample area using a custom‐built modified Howland current source, controlled by an NI 9263 voltage output module. The voltage signal from the thermopiles was measured using two two‐channel nanovoltmeters (Keithley 2182A) and a rotary switch allowed selection of which thermopiles to measure. Unless otherwise specified, all measurements were performed using channel 1 (10 mV voltage range) at a rate of six power line cycles (6 PLC), with power line synchronization and auto‐zero enabled, and the built‐in analog filter disabled, resulting in a measured sampling rate of 2.09 Hz. In addition, to reduce measurement noise, a moving average filter was applied using the number of readings as marked in the text, resulting in a reduced effective sampling frequency *f*_eff_. The RMS noise of the voltmeter with shorted input leads was measured to be 0.87 nV at an effective sampling frequency *f*_eff_ of 0.02 Hz, which is slightly better than expected based on the manufacturer's specifications. The multiprobe alignment, sensor calibration, and sample measurement were controlled using a LabVIEW program.

##### Preparation of the Hydrogel/Plastic Enclosure

The plastic ring (18 mm inner diameter, 23 mm outer diameter, and 8 mm height) was 3D printed in poly(lactic acid) (PLA) using a Monoprice Maker Select V2 printer and waterproofed by coating with a UV‐curable adhesive (Norland Optical Adhesive 81). A fully swollen polyacrylamide (PAAm) hydrogel was used as a hydrogel ring. First, a precured PAAm solution containing 10 mL of 15% w/v acrylamide (AAm; Sigma‐Aldrich), 1.5 mL of 2% w/v *N*,*N*′‐methylenebis(acrylamide) (MBAA; Sigma‐Aldrich), 165 µL of 66 mg mL^−1^ ammonium persulfate (APS; Sigma‐Aldrich), and 3.3 µL of *N*,*N*,*N*′,*N*′‐tetramethylethylenediamine (TEMED; Sigma‐Aldrich) was prepared. The precured PAAm solution was poured into a mold with the desired dimension (inner diameter: 12 mm, outer diameter: 16 mm, and height: 6.35 mm) and cured for 12 h. Once cured, the hydrogel liner was removed from the mold, swollen in deionized water for longer than a day, and inserted into the plastic ring.

## Conflict of Interest

The authors declare no conflict of interest.

## Supporting information

Supporting Information
