# Supplementary material for: A Micromachined Picocalorimeter Sensor for Liquid Samples with Application to Chemical Reactions and Biochemistry
Source: Adv Sci (Weinh). 2021 Jan 12;8(5):2003415. doi: 10.1002/advs.202003415 (PMC7927623; doi:10.1002/advs.202003415)
Supplement: Supplementary file 1 — Supporting Information [file ADVS-8-2003415-s001.pdf]

© 2021 Wiley-VCH GmbH

ADVANCED SCIENCE
Open Access

Supporting Information

for *Adv. Sci.,* DOI: 10.1002/advs.202003415

A micromachined picocalorimeter sensor for liquid samples with application to chemical reactions and biochemistry

*Jinhye Bae, Juanjuan Zheng, Haitao Zhang, Peter J. Foster, Daniel J. Needleman, Joost J. Vlassak**

Supporting Information

A micromachined picocalorimeter sensor for liquid samples with application to chemical reactions and biochemistry

Jinhye Bae†*, Juanjuan Zheng*†*, Haitao Zhang, Peter J. Foster, Daniel J. Needleman, Joost J. Vlassak**

1. Design of Sensor

The power dissipated by the sample is obtained by measuring the temperature difference between the sample and one of four references. This temperature difference is measured using thermopiles, which consist of a number of thermocouples connected in series. The voltage across a single thermocouple (Figure 1b), is given by $\Delta V_0 = (S_A - S_B)(T_h - T_c)$, where S_A and S_B are the Seebeck coefficients of the two conducting materials used in the thermocouple, T_h is the temperature of the thermocouple junction, and T_c is the temperature of the two conductors at the location where the voltage is measured. The Seebeck coefficient is a material property that is on the order of 1 - 10 μVK^{-1} for metals and 100 - 1000 μVK^{-1} for semiconductors.[1] The signal from a thermocouple with a single junction is quite small and, to improve the response, thermocouples are often connected in series to form a thermopile (Figure 1b). If a thermopile consists of n thermocouples in series, the voltage difference across the thermopile is $\Delta V = n\Delta V_0 = n(S_A - S_B)(T_h - T_c)$, *i.e.*, the response increases linearly with the number of thermocouples. The increased response of the thermopile comes at the expense of increased complexity and electrical resistance compared to a single thermocouple.

The resolution of a thermopile-based calorimeter sensor is determined by the signal-to-noise ratio of the thermopile, which depends mainly on the materials used in the thermopile and its geometry. In the following, we assume that all possible measures have been taken to eliminate extraneous sources of noise. To maximize the resolution of the calorimeter sensor,

we minimize the noise equivalent power (NEP) of the thermopile, which can be written as the ratio of the Johnson noise[2] ($V_{JN} = \sqrt{4k_BTRB}$, where k_B is Boltzmann's constant, T is temperature, R is the electrical resistance of the thermopile, and B is bandwidth) to the thermopile responsivity ($\Sigma = \frac{\Delta V}{P} = \frac{n\Delta S\Delta T}{P}$, where P is the power dissipated by the sample, ΔT is the difference between the sample and a given reference, and ΔS is the difference between the Seebeck coefficients of the materials in the thermopile):

$$NEP = \frac{V_{JN}}{\Sigma} = \frac{P\sqrt{4k_BTRB}}{n\Delta S\Delta T}. \quad \text{(S1)}$$

To evaluate the NEP, the relationship between the power P dissipated by the sample and the resulting ΔT needs to be estimated using a thermal model of the sensor. The details of the model are given in the **Analytical thermal model** section. Here, we provide a brief synopsis. The model has two components: derivation of 1) the temperature difference between the sample and the ambient, and 2) the temperature distribution along the thermopile. For typical sensor dimensions, the heat loss from the sample occurs predominantly by conduction through air; only a very small fraction of heat is lost via conduction through the thermopiles or the membrane because of their small cross-sectional area. Thus, the temperature difference between the sample and ambient is set solely by the power dissipated by the sample and the rate of heat loss to the ambient air. Assuming a temperature distribution that is approximately spherically symmetric around the sample area of the sensor, the temperature difference between sample and ambient is given by

$$\Delta T = \frac{P}{4\pi a\lambda_g}. \quad \text{(S2)}$$

Here λ_g is the thermal conductivity of air and a is the radius of the sample. This temperature difference creates a temperature profile inside the thermopile. If the thermopile is sufficiently long, the reference side of the thermopile is at ambient temperature and the thermopile generates the strongest possible signal, but it also has greater electrical resistance and more Johnson noise.

If the thermopile is short, the signal is weaker because of the smaller temperature difference, but it has lower electrical resistance and thus less noise. The temperature distribution along the length of the thermopile may be estimated by modeling the thermopile as a cooling fin that loses heat both by conduction through the fin and through air. Taking into account the temperature distribution along the length of the thermopile, the noise equivalent power can then be approximated as

$$NEP = \frac{4\pi a\lambda_g\sqrt{4k_BTRB}}{n\Delta S\left[1-exp\left(-\sqrt{\frac{\lambda_g}{kh_{TP}L_0}}l\right)\right]}, \tag{S3}$$

where l is the length of the thermopile, k is the effective thermal conductivity of the thermopile, h_{TP} is the thickness of the thermopile, and L_0 is the distance from the sensor membrane to the wall of the chamber that encloses the sensing portion of the sensor. Writing the electrical resistance of the thermopile in terms of the dimensions of the sensor, we finally find the following expression for the noise equivalent power of the sensor,

$$NEP = 16\pi\lambda_g\sqrt{k_BTB}\frac{\sqrt{\rho_A+\rho_B}}{S_B-S_A}\frac{\sqrt{lw/h_{TP}}}{1-exp\left(-\sqrt{\frac{\lambda_g}{kh_{TP}L_0}}l\right)}. \tag{S4}$$

In this expression, w is the line width of the conductors in the thermopile; ρ_A and ρ_B represent the resistivities of the thermopile materials. The first fraction in the right-hand side of Equation (S4) relates uniquely to the materials used in the thermopile; the second fraction relates solely to the dimensions of the thermopile.

2. Analytical thermal model

Assuming heat loss occurs by conduction through air only, the steady-state temperature distribution around a spherically symmetric sample that is generating heat at a rate P is given by

$$T(r) = T_A + \frac{P}{4\pi\lambda_g}\frac{1}{r}, \tag{S5}$$

where T_A is the ambient temperature, r the distance to the center of the sample, and λ_g the thermal conductivity of air. The temperature difference between the sample and its surroundings is then

$$\Delta T = \frac{P}{4\pi\lambda_g a}, \tag{S6}$$

where a the radius of the sample. To determine the temperature distribution in the thermopile, the thermopile is modeled as a heat fin, a distance L_o from a wall at temperature T_A. In the steady state, the temperature in the thermopile is then approximately

$$T(x) = T_A + \Delta T \exp\left(-\sqrt{\frac{\lambda_g}{k_{TP} h_{TP} L_o}}x\right)$$
$$= T_A + \frac{P}{4\pi\lambda_g a}\exp\left(-\sqrt{\frac{\lambda_g}{k_{TP} h_{TP} L_o}}x\right), \tag{S7}$$

where k_{TP} and h_{TP} are the effective thermal conductivity and thickness of the thermopile. Finally, the temperature drop across a thermopile of length l is approximately

$$\Delta T_{TP} = \frac{P}{4\pi\lambda_g a}\left(1 - \exp\left(-\sqrt{\frac{\lambda_g}{k_{TP} h_{TP} L_o}}l\right)\right). \tag{S8}$$

The material properties used to calculate Figure 1c are listed in **Table S1**.

Table S1. Material properties for calculating *NEP* as a function of thermopile length[3-5]

Material	Seebeck coefficient relative to platinum (μV/K)	Electrical resistivity (μΩ m)	Thermal conductivity (W/m K)
Constantan	-35	0.49	21.2
Nichrome	25	1.0	11.3
Chromel	29.8	0.71	20
Aluminum	3.5	0.0282	250
P-polysilicon*	103	22.1	31.2
N-polysilicon*	-57	8.1	31.5

3. FEM simulation

Finite element simulations were performed using the commercial software package COMSOL Multiphysics®. The parameters used in the model are listed in Table S1. To reduce the size of the model, the individual legs of the thermopiles were not resolved, but were modeled using effective material properties taking into account the geometry of the thermopiles. The effective thermal conductivity of the thermopiles was taken as 5.81W/m K in the direction of the temperature gradient, and 3.95 W/m K in the perpendicular direction, for an effective thermopile thickness of 1.25 μm.

The material properties used in the FEM model are listed in **Table S2**. The thermal conductivities of the W and Cu thin films are obtained by scaling the bulk thermal conductivity with the electrical conductivity according to the Wiedemann-Franz law.[3] For sensor 22, the measured resistance and theoretical resistance of the W heating element are 350.0 Ω and 51.7 Ω, respectively. The measured resistance and theoretical resistance of the Cu square are 0.040 Ω and 0.033 Ω, respectively.

Table S2. Material properties used in the FEM model [3, 4, 6-9]

Material	Electrical conductivity (S/m)	Thermal conductivity (W/m K)
Si_3N_4	-	3.2
Tungsten	20.0×10^6	bulk :174
		film: 26
Copper	5.998×10^7	bulk :400
		film: 330
Constantan	2.0×10^7	21.2
Nichrome	1.0×10^6	11.3
Air	-	0.026

4. Fabrication of the picocalorimetric sensors

The sensors were fabricated using standard microfabrication techniques. Figure S1 illustrates the fabrication sequence. Figure S2 shows the residual stress in the nichrome and constantan coatings as a function of Ar working gas pressure. While the majority of the processes used to fabricate the sensors are fairly standard microfabrication steps, integration of the thermopile on the Si_3N_4 membrane presents a particular challenge. Constantan and nichrome films with a thickness of 500 nm sputter deposited under typical conditions of Ar working gas pressure (3-5 mTorr) on a Si_3N_4-coated Si substrate show extensive fracture and delamination. Moreover, as soon as the sensor membrane is made freestanding, even intact constantan and nichrome films fracture because of the greatly increased energy release rate associated with a more compliant substrate[10]. Successful deposition of these films requires careful management of the Ar pressure to limit the residual stresses in the coatings (Figure S2).

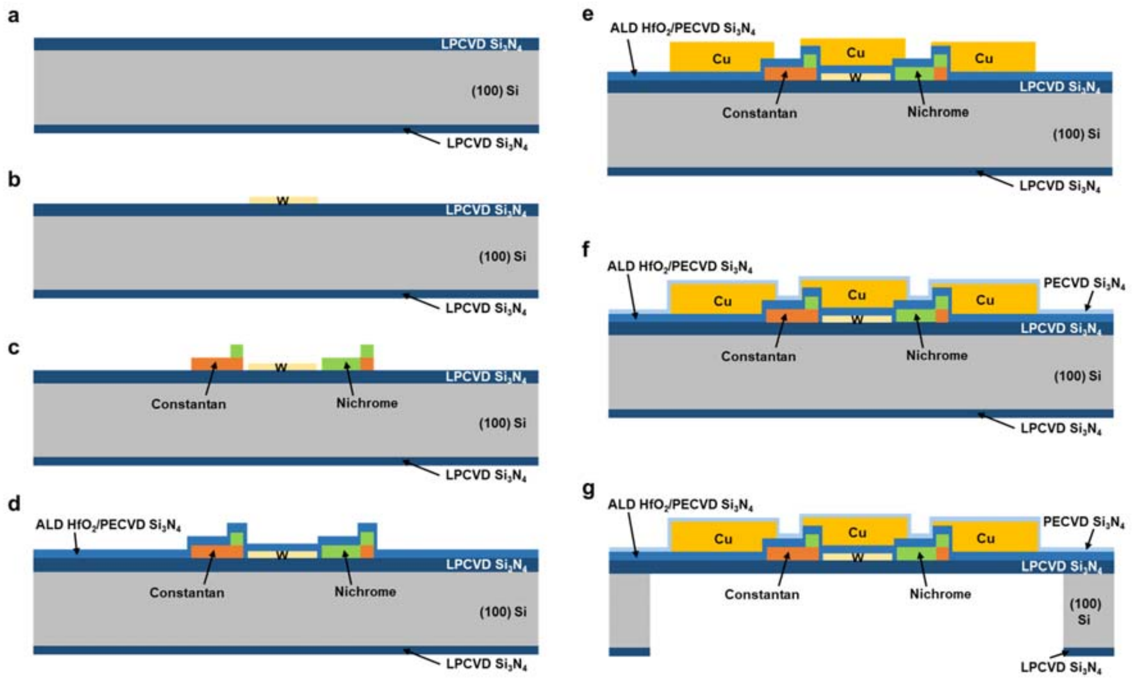

Figure S1. Fabrication flow of the calorimetric sensors. (**a**) The fabrication process starts with a (100) Si wafer with a 600 nm coating of LPCVD Si_3N_4. (**b**) A Ti/W (5/120 nm) layer is

patterned using lithography and lift-off to define the heating element. (**c**) Ti/constantan (5/500 nm) and nichrome (5/500 nm) film stacks are patterned using lithography and lift-off to define the thermopiles. (**d**) ALD HfO_2 (30 nm) and PECVD Si_3N_4 (300nm) are deposited as an electrically insulating layer. (**e**) Ti/Cu (5/1000 nm) is patterned using lithography and lift-off to define the sample and reference areas. (**f**) PECVD Si_3N_4 (100nm) is deposited as a capping layer. (**g**) The backside of the Si substrate is etched using DRIE to make the sensor freestanding.

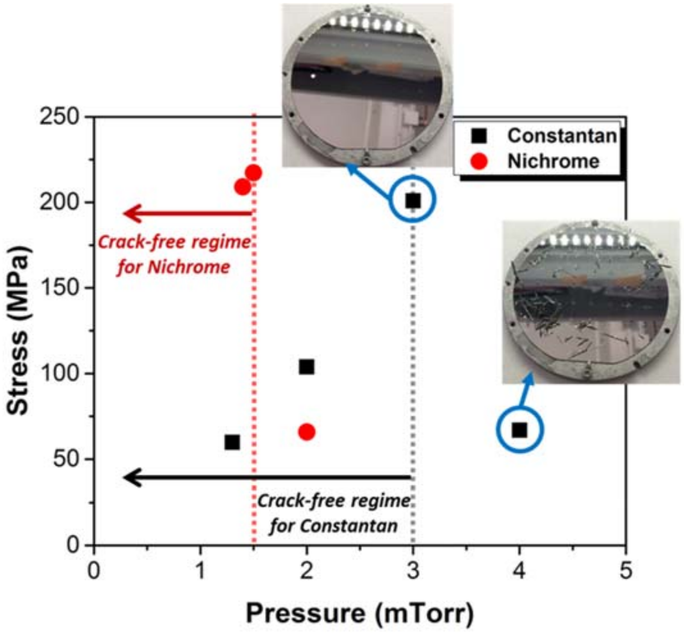

Figure S2. Residual stress in constantan and nichrome coatings. Residual stress in the constantan and nichrome coatings as a function of argon pressure during sputter deposition. Constantan is deposited without cracks at a working gas pressure of 3 mTorr, pressures of 4 mTorr or greater result in extensive cracking during deposition. Nichrome was deposited without cracking at argon pressures below 1.5 mTorr.

5. Resolution of the picocalorimetric sensors

Figure S3 shows the power spectral density (PSD) graphs for unprocessed data obtained from the voltmeter with input leads shorted, and with leads connected to just one thermopile on sensors 42 and 63. It is clear from the figure that the noise in the voltmeter and the sensors is nearly constant over the measured frequency range. Assuming that the noise generated by the thermopile is mainly Johnson noise and that the voltmeter contributes a noise of 0.87 nV, the total noise for single-thermopile measurements is given by

$$n_T = \sqrt{(0.87)^2 + 4kTRB \times 10^{18}}\, nV. \quad \text{(S9)}$$

Equation (S9) then yields a theoretical noise of 1.04 nV and 2.02 nV for sensors 42 and 63, respectively, in good agreement with the experimental values of 1.09 nV and 2.08 nV for a bandwidth of 0.02 Hz (Figure S3). Consequently, Equation (S9) was used to calculate the total single-thermopile noise for all sensors listed in Table 2. The resolution of the sensors then depends on the responsivity of the sensor and the noise in the measurements and is also listed in the table. When the four thermopiles are connected in series, the responsivity increases by a factor of four and the noise approximately by a factor of two. For instance, if the four thermopiles in sensor 63 are connected in series, the responsivity in vacuum of the sensor is 133.4 V/W and the total noise is 3.74 nV (see Table 2). Combining both results leads to a resolution in vacuum of approximately 28.0 pW. The corresponding resolution under ambient conditions is 200 pW. These values of the resolution can be further improved for slowly varying signals by accepting a smaller bandwidth.

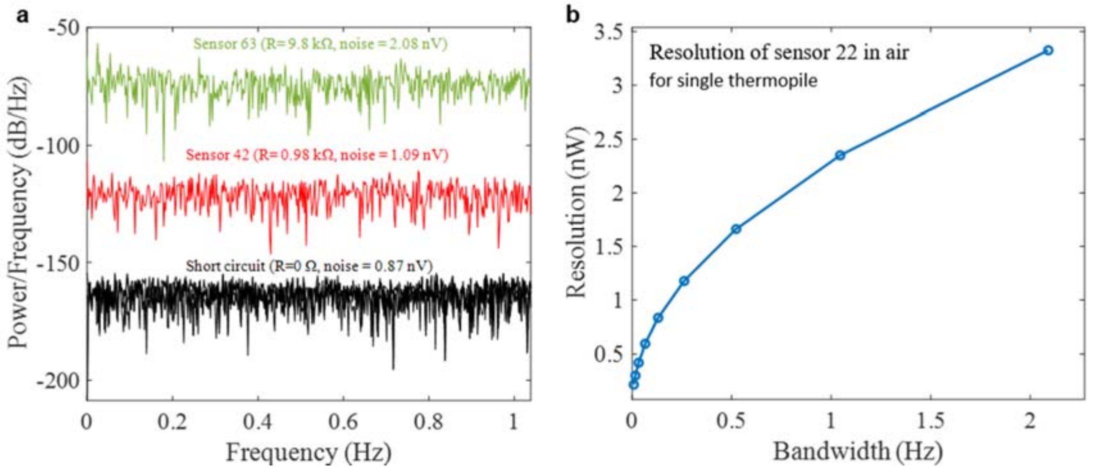

Figure S3. (a) Graphs of the power spectral density of unprocessed data obtained for the voltmeter with leads shorted, and with leads connected to sensors 42 and 63. PSD graphs for sensors 42 and 63 are offset by 40 and 80 dB/Hz, respectively. Before calculating the PSD, signal drift and offset were subtracted in the time domain. (b) Resolution in air as a function of

bandwidth for sensor 22. The resolution is shown for a single thermopile; if the four thermopiles are connected in series, the resolution improves by a factor of two.

6. Effective radius and time constant

The data in Figure 5a can be used to determine an effective radius a_{eff} that can be used to evaluate the effect of sample volume on the responsivity of the sensor. Assuming the response of the thermopiles is given by

$$\Delta V = n\Delta V_0 = n(S_A - S_B)\Delta T, \tag{S10}$$

where S_A and S_B are 25 µV/K and -35 µV/K, the Seebeck coefficient of Nichrome and Constantan, respectively. We define the effective sample radius, a_{eff}, such that the temperature difference, ΔT, between sample and reference is given by

$$\Delta T = \alpha \frac{P}{4\pi a_{eff}\lambda_g}\left(1 - exp\left(-\sqrt{\frac{\lambda_g}{kh_{TP}L_0}}\,l\right)\right), \tag{S11}$$

where α is a numerical factor that brings Equation (1) in quantitative agreement with the FEM simulations for a blank sensor and that has a value of approximately 0.35. Combining Equations (S10) and (S11) yields the following expression for a_{eff}:

$$a_{eff} = \frac{\alpha n P(S_A - S_B)}{4\pi\lambda_g \Delta V}\left(1 - exp\left(-\sqrt{\frac{\lambda_g}{kh_{TP}L_0}}\,l\right)\right). \tag{S12}$$

Equation (S12) allows direct calculation of a_{eff} as a function of sample volume V_s from the data in Figure 5a. The effective radius is then well represented by $a_{eff} = 791\ \mu\mathrm{m} + 109\ \frac{\mu\mathrm{m}}{\mu\mathrm{L}} V_s$ for the sensor used to collect the data in Figure 5a (Sensor 22). Once the effective radius is known for a given sample volume, a combination of Equations (S10) and (S11) yields the responsivity of the sensor for that sample volume.

The effective radius also elucidates how the response time of the sensor changes with sample volume and characteristics. On the assumption that heat loss happens mainly by conduction

through air and that the thermal mass of the sensor is negligible compared to that of the sample, a simple lumped thermal model results in the following expression,

$$\tau = \frac{\alpha \rho V_s c_V}{4\pi \lambda_g a_{eff}}, \tag{S13}$$

for the time constant τ of a sensor loaded with a sample of volume V_s, density ρ, and heat capacity c_V. The linear relationship between the time constant and the sample volume divided by the effective sample radius is indeed borne out in Figure S4, with a slope that is in good agreement with Equation (S13) [11, 12].

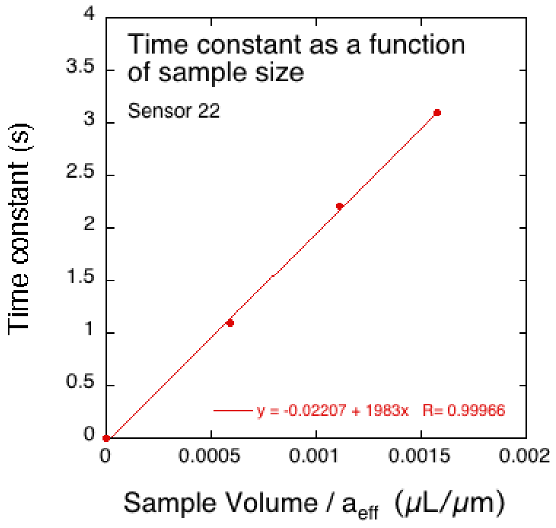

Figure S4. Time constant as a function of sample size. The time constant of Sensor 22 as a function of V_s / a_{eff} as determined from the measurements on ionic liquid droplets depicted in Figure 5a.

7. Experimental results

Figure S5 shows the evolution of the sensor signal as a function of time. Figure S6 shows the phosphate calibration curve.

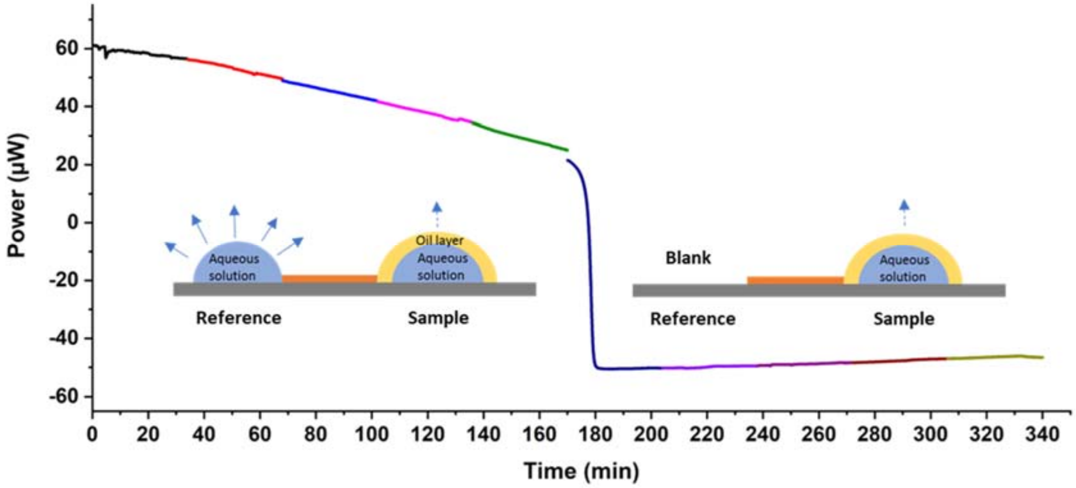

Figure S5. Effect of evaporation of aqueous solutions. Calorimetric signal obtained as a result of differential evaporation between a sample and a reference droplet. After evaporation of the droplet in the reference area, the signal changes sign as the temperature of the sample area decreases below that of the reference area. Data were obtained using a sampling frequency of 30PLC with auto-zero and power line synchronization enabled, and the built-in analog filter turned off. A 10-reading moving average digital filter was used to smooth the data.

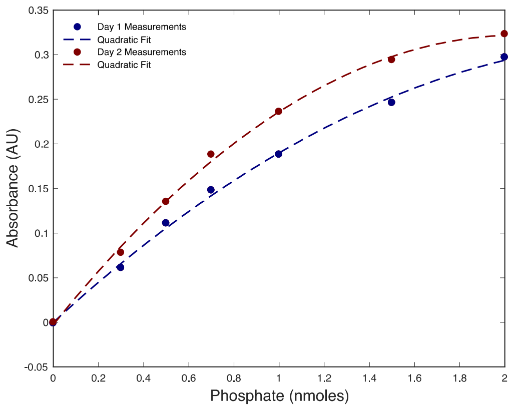

Figure S6. Phosphate calibration curve. Absorbance at 650 nm was measured for varying phosphate concentrations on each day of experiments. Measurements of absorbance vs. phosphate were fit to a quadratic function to generate calibration curves for each day.

References

[1] D. D. Pollock, *Thermoelectricity - theory, thermometry, tool*, ASTM,Philadelphia, PA, United States 1985.
[2] J. B. Johnson, Physical Review 1928, 32, 97.
[3] D. R. Lide, *CRC handbook of chemistry and physics*, Vol. 85, CRC press, 2004.
[4] R. J. Moffat, Electronics Cooling 1997, 3, 12.
[5] M. Strasser, R. Aigner, C. Lauterbach, T. Sturm, M. Franosch, G. Wachutka, Sensors and Actuators A: Physical 2004, 114, 362.
[6] P. J. McCluskey, J. J. Vlassak, Thin Solid Films 2010, 518, 7093.
[7] K. Xiao, J. M. Gregoire, P. J. McCluskey, J. J. Vlassak, Review of Scientific Instruments 2012, 83, 114901.
[8] J. Zheng, Y. Miao, H. Zhang, S. Chen, D. Lee, R. Arróyave, J. J. Vlassak, Acta Materialia 2018, 159, 320.
[9] H. Zhang, D. Lee, Y. Shen, Y. Miao, J. Bae, Y. Liu, J. Schroers, Y. Xiang, J. J. Vlassak, Acta Materialia 2018, 156, 486.
[10] J. J. Vlassak, International Journal of Fracture 2003, 119, 299.
[11] G. J. Kabo, A. V. Blokhin, Y. U. Paulechka, A. G. Kabo, M. P. Shymanovich, J. W. Magee, Journal of Chemical & Engineering Data 2004, 49, 453.
[12] Y. Huo, S. Xia, P. Ma, Journal of Chemical & Engineering Data 2007, 52, 2077.
